# Fatigue, Internet Addiction and Symptoms of Long COVID—A Cross-Sectional Study of Polish Students

**DOI:** 10.3390/jcm13123383

**Published:** 2024-06-09

**Authors:** Anna Zalewska, Monika Gałczyk

**Affiliations:** Faculty of Health Sciences, University of Lomza, 14 Akademicka St., 18-400 Lomza, Poland; monikagalczyk@onet.eu

**Keywords:** mental health, pandemic, young adults, epidemiology

## Abstract

**Introduction**: Fatigue is the most persistent symptom in patients with long COVID. Moreover, Internet addiction itself has become a pandemic long-term effect. The aim of this study was to investigate the degree of fatigue and Internet addiction in a group of students with COVID-19 and to determine the relationship between fatigue and addiction in students with and without long COVID symptoms. **Material and methods**: A cross-sectional study was conducted among 402 Polish students aged 19–26. The 183 students who had COVID-19 signaled the presence of long COVID symptoms, which corresponded to 45.5% of the surveyed group. The Modified Fatigue Impact Scale was used to assess the level of fatigue, and the Kimberly Young questionnaire was used to assess the level of Internet addiction. **Results:** 19.7% (95% c.i.: 15.9–23.9%) of the students surveyed had a moderate level of Internet dependence (Internet addiction measure value of 50 points or more). Most of them did not complain of high levels of fatigue. Higher levels of dependence and fatigue were observed in subjects with long COVID symptoms (MFIS mean value was 26.5 in this group vs. 17.7 in the others; *p* = 0.0000 ***). The higher the respondents’ level of dependence, the more they tended to feel tired (correlations were stronger in those with long COVID symptoms: *r*_S_ = 0.23; *p* = 0.0017 **). **Conclusions:** In view of the results obtained, the study presented here has the potential to contribute to the international debate on the long-term health consequences of the COVID-19 pandemic and strategies to address them. The study provides data that may be useful in the development of educational and health policies that target the psychophysical well-being of patients with long COVID symptoms. This process should be considered as a long-term endeavor.

## 1. Introduction

In today’s world, the development of information technology and, in particular, all devices with Internet access, has become of interest not only to the users themselves, but also to researchers. This development has an impact on many areas of life, from business to social interaction, for both adults and children. Quick and easy access to entertainment using mobile networks has led to more frequent use of the Internet. As a result, more and more people have become addicted to the Internet in recent years. Certainly, this phenomenon is closely linked to the Coronavirus disease 2019 (COVID-19) pandemic and, in a way, a consequence of it that affects daily life [1,2].

The concept of Internet addiction is compared to the traditional addiction to certain psychoactive substances, characterized by a lack of control and withdrawal symptoms [3]. Statistics show that almost 4 billion people used the Internet in 2018 [4,5,6,7,8]. The vast majority of these were adolescents Numerous research have been conducted and published in the literature to confirm the detrimental consequences of mobile phone addiction on biopsychosocial health [9,10,11,12].

Fatigue is a rather complex phenomenon that is defined differently in various disciplines such as psychology, management or medicine [13,14]. In medicine, the concept of fatigue itself is not considered a single disease and is quite a common phenomenon. It is defined as a normal reaction of the body to prolonged activity, after which the body needs to recover [15,16,17]. Some lifestyle factors can also affect fatigue, such as sleep quality, irregular sleep or excessive nocturnal activity [18,19,20].

Coronavirus disease 2019 (COVID-19) is an infectious respiratory disease with droplet transmission caused by infection with the SARS-CoV-2 virus, which can lead to respiratory dysfunction and physical and mental deterioration [21]. After the acute phase, this can develop into ‘post-COVID-19 syndrome (PCS)’ or ‘long COVID’, which is defined as a syndrome of physical and subjective symptoms that cannot be explained by other diagnoses and that persists for more than 12 weeks after infection with COVID-19 [21]. It is estimated that 10% of people who test positive for COVID-19 have prolonged COVID syndrome. Due to the large number of people who have or have had COVID-19, PCS is a serious global problem [21].

And although there is no doubt that the Internet plays a key role in the lives of most people around the world as a means of information and communication in all areas of life, excessive use can have negative health consequences. Research during the pandemic found a link between the risk of hikikomori (extreme social isolation) and changes in Internet use among young people aged 16–24 during COVID-19 pandemic restrictions. As it turned out, online social interaction may be a way to reduce the risk of hikikomori in post-COVID-19 societies [22,23].

As the results of the literature review show, addictive behaviour and fatigue should be continuously monitored in the post-pandemic period, especially in people with long-lasting COVID symptoms.

The purpose of this study was to determine the relationship between the degree of fatigue and internet addiction in students with and without chronic COVID-19 symptoms, as well as the degree of fatigue and Internet addiction in a group of students suffering from COVID-19.

## 2. Materials and Methods

### 2.1. Participants and Procedure

Between November and December 2023, the authors conducted an online cross-sectional survey among Polish students with COVID-19. The researchers used social media and e-learning platforms to distribute an optional, anonymous survey. Students were informed about the purpose of the survey and could unsubscribe from participation at any time. Student status, age ≥ 18, a history of COVID-19 disease (verified by a positive COVID-19 nasopharyngeal polymerase chain reaction (PCR) test), and agreement to take part in the study were the inclusion criteria. Students with musculoskeletal injuries, chronic musculoskeletal, cardiovascular and respiratory diseases, fibromyalgia or chronic fatigue syndrome were excluded from the study.

**Figure 1:**
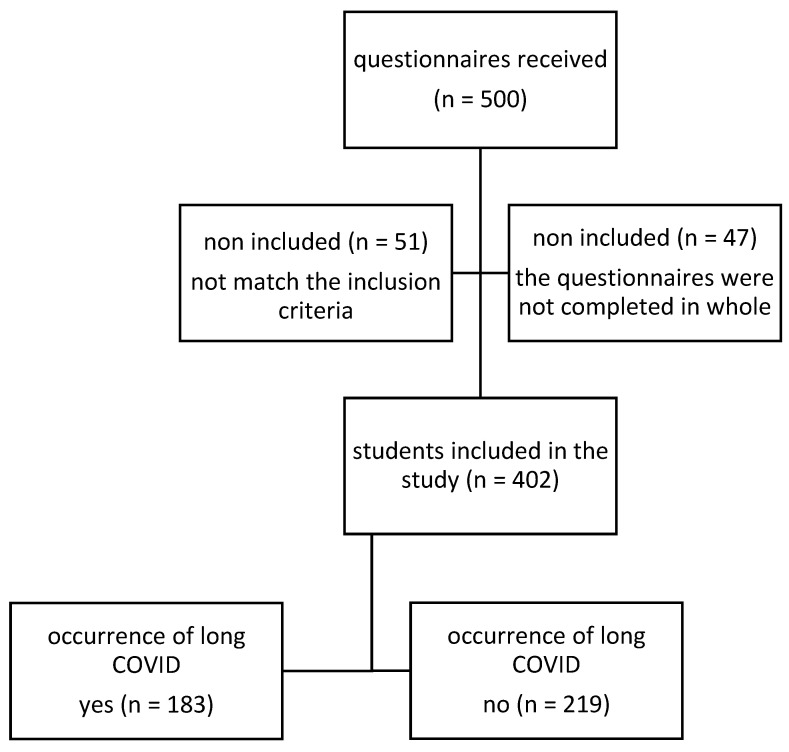
Flow chart of students selection.

A definition of long COVID syndrome was provided in the questionnaire along with a list of its characteristic symptoms, which included general symptoms like fever, exhaustion or fatigue that makes daily living difficult, and respiratory and cardiac symptoms like shortness of breath, cough, chest pain, rapid heartbeat, or palpitations; neurological symptoms like headaches, difficulty concentrating, dizziness when standing up, tingling, changes in taste or smell, depression, or anxiety; gastrointestinal symptoms like diarrhea, abdominal pain; and other symptoms like joint or muscle pain, skin rash, and changes in the menstrual cycle [21]. Out of the 500 students that took part in the study, 98 questionnaires could not be analyzed either the respondents did not match the inclusion criteria or the questionnaires were not completed in whole. Finally, completed questionnaires were provided by 402 (Figure 1). The University of Medical Sciences in Bialystok’s Senate Committee on Ethics in Scientific Research granted the authors permission to carry out the study (KB/18/2020.2021).

### 2.2. Methods for Assessing Fatigue

With the use of the Modified Fatigue Impact scale the level of fatigue was evaluated [24], which consists of 3 parts (F-1 for the impact of fatigue on physical performance; F-2 for the impact of fatigue on cognitive functions; F-3 for psychosocial functions). The MFIS examines fatigue levels over the past 4 weeks, with an overall score ranging from 21 to 105. The greater the impact of weariness on functioning, the higher the score. The articles report a Cronbach’s alpha value of >0.7 [25].

### 2.3. Methods for Assessing Internet Addiction

The Kimberly Young questionnaire was used to assess the degree of Internet addiction [26]. It includes 20 questions covering various aspects of how frequently people use the Internet. On a 5-point rating system, with 1 denoting never and 5 denoting always, respondents indicate their responses. The entire score’s summed metric falls between 20 and 100 points. Three categories—low, medium, and high—are assigned to the score (which spans the ranges 20–49, 50–79, and 80–100, respectively). The articles report a Cronbach’s alpha value of greater than 0.7 [27].

### 2.4. Statistical Methods

Statistica v.13 (TIBCO Software Inc., 2017, Palo Alto, CA, USA) was used for the statistical study. The mean and standard deviation were used to present the descriptive data for the different study variables. The Mann-Whitney test was used to assess the significance of differences in fatigue levels between men and women. The investigation of the relationship between Internet addiction and fatigue levels in individuals with and without long COVID was performed using the non-parametric Spearman’s rank correlation coefficient. A significance level of *p* < 0.05 was taken into account for all statistical analyses.

## 3. Results

### 3.1. Respondent Population

The analysis concerned 402 students (242 women and 160 men) aged 19–26 years who had COVID-19. Women predominated in the study population (probably due to the specificity of the subjects analyzed.

With a standard deviation of 1.2 years, the average age of the respondents was 21.5 years. The 183 respondents with COVID-19, i.e., 45.5% of the sample, reported that they suffered from post-COVID-19 syndrome (PCS) symptoms.

Most of the respondents were studying medicine (22.4%), administration (16.2%), philology (10.4%) and pedagogy (7.7%). Overall, only 0.4% of respondents studied agriculture, forestry, military and maritime studies.

### 3.2. Level of Internet Addiction

The distribution of Internet addiction severity throughout the whole study population and by student gender is displayed in Table 1. There is no difference in the extent of Internet addiction between men and women (*p* = 0.3659). The score in the upper quartile indicates that the vast majority of students surveyed would be classified as having a low level of Internet addiction (<50 points).

**Figure 2:**
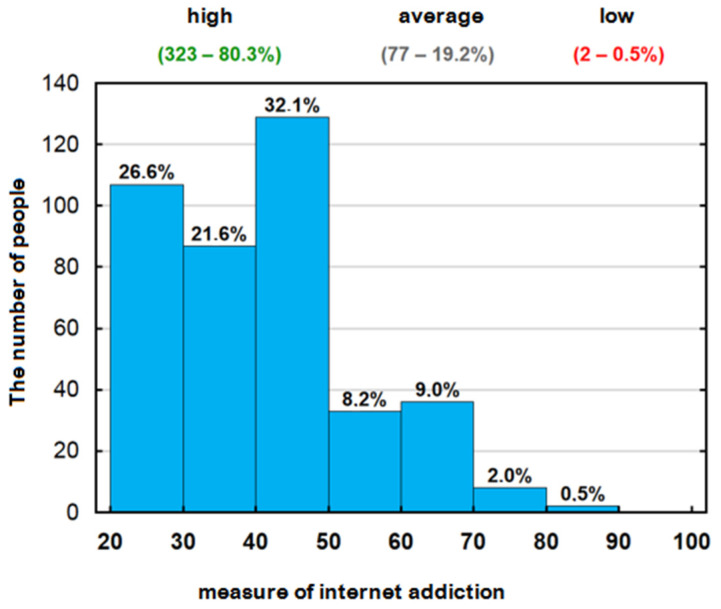
Distribution of the Internet addiction measure after grouping the values into intervals with a spread of 10 points.

Figure 2 shows the distribution of the Internet addiction measure after grouping the values into ranges with a spread of 10 points. As can be seen, 19.7% (95% c.i.: 15.9–23.9%) of the students have an Internet addiction measure value of 50 points or more, indicating a medium level of addiction. Occasionally (0.5%) there is a high level, corresponding to a value of 80 points or more.

### 3.3. Internet Addiction and Long COVID Symptoms

The co-occurrence of Internet addiction and long COVID symptoms were examined (Table 2). People who report the presence of long COVID symptoms have a significantly higher level of Internet addiction (on average by more than 5 points). This correlation exists for both sexes.

### 3.4. Fatigue Level

The table (Table 3) shows the level of fatigue, as assessed by the MFIS measure. 

**Figure 3:**
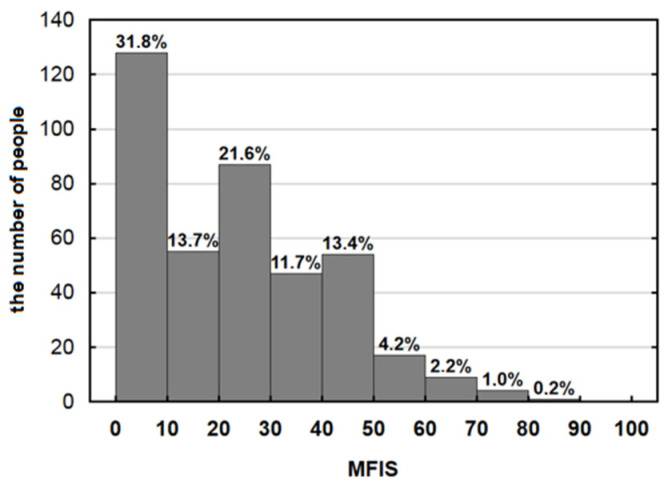
Measures of overall fatigue.

The distribution of the MFIS measure of overall fatigue is shown in the following graph (Figure 3), after grouping the values into intervals with a range of 10 points. The strong asymmetry of the distribution of this measure is evident, with very low scores dominating, indicating that most students surveyed did not complain of high levels of fatigue.

There were no statistically significant differences in the level of fatigue declared by male and female students (Table 4).

### 3.5. Internet Addiction and Fatigue Levels

Correlations between Internet addiction and fatigue were examined (Table 5). 

**Figure 4:**
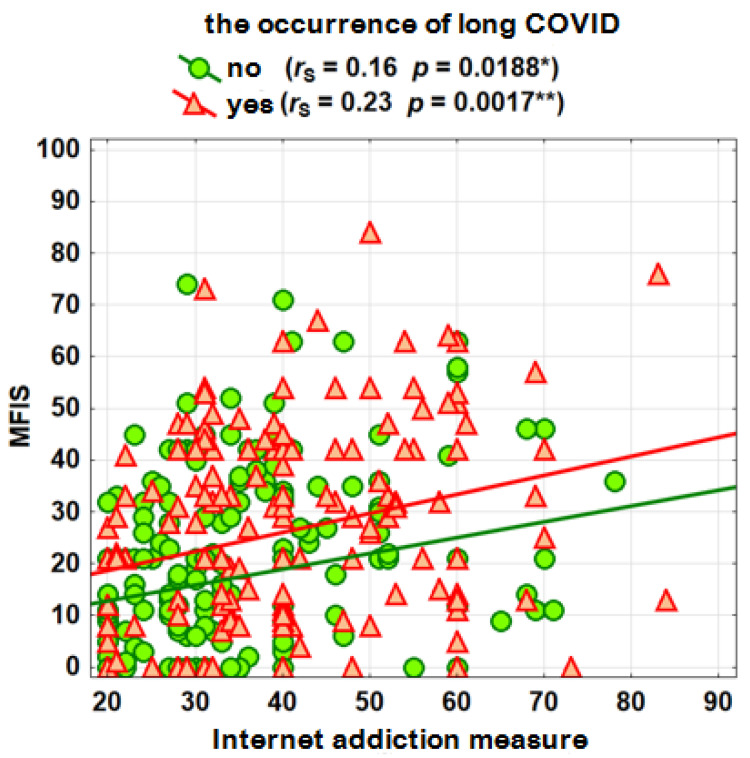
Correlations between the holistic fatigue measure and the Internet addiction measure. *p* < 0.05 (*), *p* < 0.01 (**).

As the previous analysis shows that long COVID differentiates the level of both phenomena, the analysis was conducted separately for both groups. As can be seen, there are statistically significant, albeit weak, correlations between fatigue and Internet addiction. The higher the level of Internet addiction, the greater the tendency to experience fatigue. These relationships, although generally weak, are slightly stronger among those with long COVID. The graph (Figure 4) shows the relationship between the measures of Internet addiction and holistic fatigue.

The simple regressions shown in the graph suggest that for the same level of Internet addiction, people with long COVID have higher overall fatigue, on average by about 10 points.

It was investigated whether the presence of long COVID symptoms was associated with higher levels of fatigue (Table 6). Those reporting this condition reported significantly higher levels of fatigue—the differences are highly statistically significant, with an overall level of fatigue of almost 10 points (on average).

Although sex had no effect on the scores in the study group, the association between the occurrence of long COVID and fatigue was investigated, also taking this additional factor into account (Table 7). It appears that the relationship between long COVID and fatigue is a domain of women. In this group, the occurrence of long COVID is associated with an overall fatigue level that is almost 12 points higher, while in men such a difference is around 4.5 points. Statistically significant differences are only found in the group of women.

## 4. Discussion

Fatigue after an acute viral illness is a major problem that complicates the clinical course of epidemic and non-epidemic viral infections [28]. According to a review of the literature, it is also the symptom with the longest persistence in patients with long COVID [29,30]. In addition, fatigue does not usually seem to disappear after the usual six-month recovery period after COVID-19 [29]. Addictive behaviour is an important determinant of health and tends to increase during public crises, which the COVID-19 pandemic undoubtedly was [31,32]. Internet addiction became a long-term effect of the COVID-19 pandemic [33].

In the early days of the pandemic, Internet use among young people increased almost worldwide [34,35]. Over time, the overall trend of addictive Internet use among students declined [32]. This is confirmed by our own results. Approximately 20% of students had a medium level of addiction. In contrast, the scores obtained for adolescents from Bangladesh, India and Lebanon are more alarming and indicate that students from this part of the world are still facing serious problems related to Internet addiction even after the pandemic [36].

In our study, there was no sex difference in the level of Internet addiction (*p* = 0.3659), which is confirmed by data from the literature [37]. Only the presence of long COVID symptoms was influenced by a higher level of addiction.

The aspect of fatigue during and after a pandemic has been studied in other countries [28,38]. In the study group of students, the majority of respondents did not complain of high levels of fatigue. In general, most studies available in the literature confirm the correlation of fatigue with age. The prevalence and severity of fatigue was found to be higher in older people than in younger age groups (e.g., students) [29]. No statistically significant differences were found in the level of fatigue reported by male and female students—the sex of the subjects did not differentiate the distribution of MFIS measures. Some of the information in the literature is consistent with the data obtained, where the levels of fatigue were similar in males and females [39,40,41,42].

Statistically significant correlations were found between fatigue and Internet addiction in the research group. The greater the level of Internet dependence, the higher the tendency towards more fatigue. This correlation was already established before the pandemic [19,43], and during the pandemic It was discovered that using social media to look up information about the pandemic was linked to noticeably higher levels of weariness. [44]. In addition, an excess of ‘pandemic content’ on social media contributed to higher levels of fatigue [45]. Literature-based data also unequivocally show that students who are addicted to the Internet are far more likely to experience anxiety, weariness, and depressed symptoms, all of which negatively impact their academic performance. [46]. Similar correlations have been observed between problematic smartphone use and fatigue [47]. The results of the literature review also strongly suggest that Internet addiction can have a dual effect on physical and mental fatigue [19].

The associations of Internet addiction and fatigue were slightly stronger among those with long COVID. At the same level of Internet addiction, those with long COVID showed higher overall fatigue, on average by about 10 points. The literature review shows that the severity of perceived fatigue can be increased by the simultaneous presence of neuropsychiatric disorders. This correlation also applies to people with long COVID [31].

The prevalence of fatigue in patients with long COVID is significantly higher in the literature than in the control group. This applies to students [48] as well as other population groups [49,50,51]. These data are consistent with the authors’ findings that students who had long COVID had significantly higher levels of fatigue—the differences were highly statistically significant, with an overall fatigue measure of almost 10 points (on average). In our study, we also found that the association between long COVID and fatigue was the domain of women. In this group, the presence of long COVID was associated with an overall fatigue level almost 12 points higher, while in men such a difference was around 4.5 points. Statistically significant differences were only found in the group of women. These results are in line with a global trend where greater fatigue was observed in the female population who contracted COVID-19 during [28] and after the pandemic [52]. This could be caused by hormonal changes, among other things, which prolong the medical condition in the acute inflammatory phase long after the infection has healed., and may also be due to higher IgG antibody levels that provide symptom relief, but when present, these high IgG antibody levels persist and cause physical symptoms [29]. It has also been observed in the literature that long COVID symptoms are more common in women [31].

The research presented here has its constraints. The first is its cross-sectional nature, as it does not come up with clear causal evidence for the observed relationships and only assesses participants at one point in time. It is also potentially susceptible to selection bias, i.e., the characteristics of the students studied may not be representative of the population as a whole. People who observed COVID symptoms in themselves for a long time and suffered from fatigue may have been more likely to participate in the study. 

A second drawback of the study is that specific clinical and social factors, such as time elapsed after infection and COVID-19 vaccination status, which may have influenced the onset of long-lasting COVID symptoms, were not taken into account. Self-report questionnaires were used as study instruments, which could affect the quality of responses. The presented study is also the first to describe the level of fatigue and Internet addiction among Polish students with long COVID, which, according to the authors, is the most important strength of the manuscript. The authors also obtained more data on the prevalence of fatigue and Internet addiction among young people with long COVID thanks to their study.

## 5. Conclusions

The study presented here can contribute to the global debate on the long-term health effects of the COVID-19 pandemic and measures to contain it.

The authors found that higher levels of dependence and fatigue were observed in students with long COVID symptoms. The higher the subjects’ dependency level, the more they tended to be fatigued (the correlations were stronger in those with long COVID symptoms). Accordingly it provides data that can be useful in the development of educational and health policies aimed at the psychophysical well-being of patients with symptoms of long COVID syndrome. The process should be seen as a long-term endeavour that will enable the introduction of pre-standardized early intervention methods in emergency situations. The authors also recommend that healthcare providers pay more attention to patients with symptoms of long COVID syndrome, as they are at risk of developing fatigue.

## Figures and Tables

**Table 1:** Measures of Internet addiction in the entire study population by sex.

Sex	Measure of Internet Addiction					
	*N*	Mean	Median	Std. Dev.	Lower Quartile	Upper Quartile
Woman	242	38.7	40	12.9	30	40
Man	160	37.6	38.5	13.6	27	44.5
Total	402	38.3	40	13.2	29	42

**Table 2:** Internet addiction and long COVID symptoms by gender.

Occurrence Long COVID-19	Measure of Internet Addiction					
	*N*	Mean	Median	Std. Dev.	Lower Quartile	Upper Quartile
**Woman (** *p* = 0.0123 *)						
**not**	130	36.8	39.5	12.7	28	40
**yes**	112	41.0	40	12.9	32	50.5
**Man (** *p* = 0.0018 **)						
**not**	89	34.6	33	12.1	24	40
**yes**	71	41.4	40	14.6	31	50

*p* < 0.05 (*), *p* < 0.01 (**).

**Table 3:** Level of fatigue in the study group.

MFIS	Mean	Median	Std. Dev.	Lower Quartile	Upper Quartile
F-1 (physical)	8.8	9	7.8	1	14
F-2 (cognitive)	10.9	10	9.1	2	18
F-3 (psychosocial)	2.0	2	1.9	0	4
MFIS (comprehensive)	21.7	21	18.5	5	35

**Table 4:** Gender and distribution of MFIS measures.

MFIS	Sex										*p*
	Woman (*N* = 242)					Man (*N* = 160)					
	Mean	Me	Std. Dev.	Q_1_	Q_3_	Mean	Me	Std. Dev.	Q_1_	Q_3_	
F-1 (physical)	8.6	9	7.6	0	14	9.2	9	8.2	3	14	0.6254
F-2 (cognitive)	10.5	10	8.9	0	18	11.4	10	9.5	4	17	0.4523
F-3 (psychosocial)	2.0	2	1.9	0	4	2.0	2	1.9	0	3	0.6448
MFIS (comprehensive)	21.1	21	18.0	0	36	22.6	21	19.3	7	33.5	0.4481

**Table 5:** Associations between Internet addiction and fatigue levels.

MFIS	Occurrence of Long COVID-19	
	Not	Yes
	Measure of Internet Addiction	
F-1 (physical)	0.17 (*p* = 0.0140 *)	0.22 (*p* = 0.0024 **)
F-2 (cognitive)	0.14 (*p* = 0.0328 *)	0.23 (*p* = 0.0020 **)
F-3 (psychosocial)	0.18 (*p* = 0.0079 **)	0.25 (*p* = 0.0006 ***)
MFIS (comprehensive)	0.16 (*p* = 0.0188 *)	0.23 (*p* = 0.0017 **)

*p* < 0.05 (*), *p* < 0.01 (**), *p* < 0.001 (***).

**Table 6:** Long COVID and level of fatigue.

MFIS	Occurrence of Long COVID										*p*
	No (*N* = 219)					Yes (*N* = 183)					
	Mean	Me	Std. Dev.	Q_1_	Q_3_	Mean	Me	Std. Dev.	Q_1_	Q_3_	
F-1 (physical)	7.1	5	7.2	0	11	11.0	9	8.0	4	18	0.0000 ***
F-2 (cognitive)	9.1	8	8.9	0	16	13.1	11	9.0	6	20	0.0000 ***
F-3 (psychosocial)	1.6	1	1.8	0	2	2.5	2	1.9	1	4	0.0000 ***
MFIS (comprehensive)	17.7	14	17.5	0	29	26.5	21	18.6	11	42	0.0000 ***

*p* < 0.001 (***).

**Table 7:** Long COVID and fatigue levels by gender.

MFIS	Occurrence of Long COVID										*p*
	Not					Yes					
	Mean	Me	Std. Dev.	Q_1_	Q_3_	Mean	Me	Std. Dev.	Q_1_	Q_3_	
	**Woman**										
F-1 (physical)	6.2	4	6.6	0	11	11.4	10.5	7.7	5	18	0.0000 ***
F-2 (cognitive)	8.1	6	8.4	0	16	13.4	14	8.7	7	20	0.0000 ***
F-3 (psychosocial)	1.4	1	1.7	0	2	2.6	2	1.9	1.5	4	0.0000 ***
MFIS (comprehensive)	15.7	11.5	16.2	0	29	27.4	29	17.9	13	42	0.0000 ***
	**Man**										
F-1 (physical)	8.2	8	7.9	2	10	10.4	9	8.5	4	18	0.0803
F-2 (cognitive)	10.4	10	9.4	2	16	12.5	10	9.5	5	20	0.1356
F-3 (psychosocial)	1.9	2	1.9	0	3	2.2	2	1.9	0	4	0.1642
MFIS (comprehensive)	20.6	21	18.9	4	30	25.2	21	19.6	9	42	0.1149

*p*-test probability value calculated with the Mann-Whitney test, *p* < 0.001 (***).

## Data Availability

The data that support the findings of this study and the relevant questionnaires are available from the corresponding author, [A.Z.], upon reasonable request.

## References

[1] Olson J.A., Sandra D.A., Colucci É.S., Al Bikaii A., Chmoulevitch D., Nahas J., Raz A., Veissière S.P. (2022). Smartphone addiction is increasing across the world: A meta-analysis of 24 countries. Comput. Hum. Behav..

[2] Horwood S., Anglim J., Mallawaarachchi S.R. (2021). Problematic smartphone use in a large nationally representative sample: Age, reporting biases, and technology concerns. Comput. Hum. Behav..

[3] D’Angelo J., Moreno M.A. (2020). Screening for Problematic Internet Use. Pediatrics.

[4] Alam S., Hashim N.M.H.N., Ahmad M., Wel C.A.C., Nor S.M., Omar N.A. (2014). Negative and positive impact of internet addiction on young adults: Empericial study in Malaysia. Intang. Cap..

[5] Zenebe Y., Kunno K., Mekonnen M., Bewuket A., Birkie M., Necho M., Seid M., Tsegaw M., Akele B. (2021). Prevalence and associated factors of internet addiction among undergraduate university students in Ethiopia: A community university-based cross-sectional study. BMC Psychol..

[6] Cheng C., Li A.Y.L. (2014). Internet addiction prevalence and quality of (real) life: A meta-analysis of 31 nations across seven world regions. Cyberpsychol. Behav. Soc. Netw..

[7] Aguilar-Latorre A., Navarro C., Olivan-Blazquez B., Gervilla E., Botaya R.M., Calafat-Villalonga C., Serrano-Ripoll M.J. (2020). Effectiveness and cost-effectiveness of a lifestyle modification programme in the prevention and treatment of subclinical, mild and moderate depression in primary care: A randomised clinical trial protocol. BMJ Open.

[8] Masaeli N., Farhadi H. (2021). Prevalence of Internet-based addictive behaviors during COVID-19 pandemic: A systematic review. J. Addict. Dis..

[9] Chen Y., Zhu J., Zhang W. (2021). Reciprocal longitudinal relations between peer victimization and mobile phone addiction: The explanatory mechanism of adolescent depression. J. Adolesc..

[10] Liu Q., Zhang D., Yang X., Zhang C., Fan C., Zhou Z. (2018). Perceived stress and mobile phone addiction in Chinese adolescents: A moderated mediation model. Comput. Hum. Behav..

[11] Lian S.L., Sun X.J., Zhou Z.K., Fan C.Y., Niu G.F., Liu Q.Q. (2018). Social networking site addiction and undergraduate students’ irrational procrastination: The mediating role of social networking site fatigue and the moderating role of effortful control. PLoS ONE.

[12] Lian S., Sun X., Niu G., Yang X., Zhou Z., Yang C. (2021). Mobile phone addiction and psychological distress among Chinese adolescents: The mediating role of rumination and moderating role of the capacity to be alone. J. Affect. Disord..

[13] Finsterer J., Mahjoub S.Z. (2014). Fatigue in Healthy and Diseased Individuals. Am. J. Hosp. Palliat. Med..

[14] Lee A.R., Son S., Kim K.K. (2016). Information and communication technology overload and social networking service fatigue: A stress perspective. Comput. Hum. Behav..

[15] Fu S., Li H., Liu Y., Pirkkalainen H., Salo M. (2020). Social media overload, exhaustion, and use discontinuance: Examining the effects of information overload, system feature overload, and social overload. Inf. Process. Manage..

[16] Azzolino D., Cesari M. (2022). Fatigue in the COVID-19 pandemic. Lancet Healthy Longev..

[17] Garrigues E., Janvier P., Kherabi Y., Le Bot A., Hamon A., Gouze H., Nguyen Y. (2020). Post-discharge persistent symptoms and health-related quality of life after hospitalization for COVID-19. J. Infect..

[18] Kizilbash S.J., Ahrens S.P., Bruce B.K., Chelimsky G., Driscoll S.W., Harbeck-Weber C., Fischer P.R. (2014). Adolescent fatigue, POTS, and recovery: A guide for clinicians. Curr. Probl. Pediatr. Adolesc. Health Care.

[19] Liang S., Ren Z., Yang G. (2022). Cross-sectional and prospective association between internet addiction and risk of fatigue among Chinese college students. Medicine.

[20] Bener A., Yildirim E., Torun P., Çatan F., Bolat E., Alıç S., Griffiths M.D. (2018). Internet addiction, fatigue, and sleep problems among adolescent students: A large-scale study. Int. J. Ment. Health Addict..

[21] de Sire A., Moggio L., Marotta N., Agostini F., Tasselli A., Drago Ferrante V., Curci C., Calafiore D., Ferraro F., Bernetti A. (2022). Impact of Rehabilitation on Fatigue in Post-COVID-19 Patients: A Systematic Review and Meta-Analysis. Appl. Sci..

[22] Gavin J., Brosnan M. (2022). The Relationship Between Hikikomori Risk and Internet Use During COVID-19 Restrictions. Cyberpsychology Behav. Soc. Netw..

[23] https://www.cdc.gov/coronavirus/2019-ncov/long-term-effects/index.html.

[24] Gruszczak A., Bartosik-Psujek H., Pocińska K., Stelmasiak Z. (2009). Validation analysis of selected psychometric features of Polish version of modified fatigue impact scale–preliminary findings. Neurol. Neurochir. Pol..

[25] Meca-Lallana V., Brañas-Pampillón M., Higueras Y., Candeliere-Merlicco A., Aladro-Benito Y., Rodríguez-De la Fuente O., Ballesteros J. (2019). Assessing fatigue in multiple sclerosis: Psychometric properties of the five-item modified fatigue impact scale (MFIS-5). Mult. Scler. J. –Exp. Transl. Clin..

[26] Majchrzak P., Ogińska-Bulik N., Ogińska-Bulik N. (2006). Zachowania ryzykowne związane z cyberprzestrzenią. Zachowania Ryzykowne Dzieci i Młodzieży. Polska Adaptacja Internet Addiction Test.

[27] Salarvand S., Bagheri Z., Keshvari M., Dalvand P., Gheshlagh R.G., Keshvari M. (2018). The Prevalence of Internet Addiction and Its Relations to the Self Esteem and Life Satisfaction in Students of a Medical University. Acta Med. Iran..

[28] Khatib S., Sabobeh T., Habib A., John S., Gomez R., Sivasankar S., Masoud A. (2023). Post-COVID-19 fatigue as a major health problem: A cross-sectional study from Missouri, USA. Ir. J. Med. Sci..

[29] Salari N., Khodayari Y., Hosseinian-Far A., Zarei H., Rasoulpoor S., Akbari H., Mohammadi M. (2022). Global prevalence of chronic fatigue syndrome among long COVID-19 patients: A systematic review and meta-analysis. BioPsychoSoc Med..

[30] Babicki M., Kołat D., Kapusta J., Kałuzińska-Kołat Ż., Jankowski P., Mastalerz-Migas A., Chudzik M.P. (2023). Prevalence and assessment of risk factors among Polish adults with post-COVID-19 syndrome: A 12-month follow-up study. Pol. Arch. Med. Wewnętrznej.

[31] Paradowska-Nowakowska E., Łoboda D., Gołba K.S., Sarecka-Hujar B. (2023). Long COVID-19 Syndrome Severity According to Sex, Time from the Onset of the Disease, and Exercise Capacity—The Results of a Cross-Sectional Study. Life.

[32] Song Y.X., Huang Y.C., Li Y.Y., Bao Y.P., Zhou G.D., Lu L., Sun Y. (2023). Risk factors for poor progression of addictive internet use across different COVID-19 periods in China. Am. J. Addict..

[33] Ran M.S., Xiao Y., Rohlof H. (2024). Editorial: The impact of COVID-19 on internet addiction, suicidal behavior, and study behavior in adolescents in various cultural contexts. Front. Psychiatry.

[34] Putra P.Y., Fithriyah I., Zahra Z. (2023). Internet Addiction and Online Gaming Disorder in Children and Adolescents During COVID-19 Pandemic: A Systematic Review. Psychiatry Investig..

[35] Daglis T. (2021). The Increase in Addiction during COVID-19. Encyclopedia.

[36] Siddik M.A.B., Ali A., Miah S., Hasan M., Ahmed M., Sunna T.C. (2024). Psychological disorders among college going students: A post COVID-19 insight from Bangladesh. J. Affect. Disord. Rep..

[37] Sehrawat C., Neelam Pandey N. (2022). Internet Addiction and Social Anxiety Among Adolescents Post Covid in Digital World. Int. J. Indian Psychol..

[38] El Sayed S., Shokry D., Gomaa S.M. (2021). Post-COVID-19 fatigue and anhedonia: A cross-sectional study and their correlation to post-recovery period. Neuropsychopharmacol. Rep..

[39] Moreno-Pérez O., Merino E., Leon-Ramirez J.M., Andres M., Ramos J.M., Arenas-Jiménez J., COVID19-ALC research group (2021). Post-acute COVID-19 syndrome. Incidence and risk factors: A Mediterranean cohort study. J. Infect..

[40] Chopra V., Flanders S.A., O’Malley M., Malani A.N., Prescott H.C. (2021). Sixty-day outcomes among patients hospitalized with COVID-19. Ann. Intern. Med..

[41] Daher A., Balfanz P., Cornelissen C., Müller A., Bergs I., Marx N., Müller T. (2020). Follow up of patients with severe coronavirus disease 2019 (COVID-19): Pulmonary and extrapulmonary disease sequelae. Respir. Med..

[42] Bielecka-Dabrowa A., Sakowicz A., Gryglewska-Wawrzak K., Kapusta J., Banach M., Jankowski P., Chudzik M. (2024). The Effect of Sex on the Risk of Long-COVID and Cardiovascular Complications in Healthy Patients without Comorbidities: Data from a Polish Long-COVID Cardiovascular (PoLoCOV-CVD) Study. J. Clin. Med..

[43] Mukhlif H.H., Younis N.M. (2022). Evaluation of the association between internet addiction and fatigue among undergraduate students at universities in Mosul city, Iraq: A cross-sectional study. RMJ.

[44] Torales J., González-Urbieta I., Barrios I., Waisman-Campos M., Terrazas-Landivar A., Viola L., Ventriglio A. (2023). “Pandemic Fatigue” in South America: A Multi-Center Report from Argentina, Bolivia, Paraguay, Peru, and Uruguay. Brain Sci..

[45] Chen M., Yu W., Cao X. (2023). Experience Pandemic Fatigue? Social Media Use May Play a Role: Testing a Model of Pandemic Fatigue Development from a Social Media Perspective. Health Commun..

[46] Sayed M., Naiim C.M., Aboelsaad M., Ibrahim M.K. (2022). Internet addiction and relationships with depression, anxiety, stress and academic performance among Egypt pharmacy students: A cross-sectional designed study. BMC Public Health.

[47] Zhang C., Zeng P., Tan J., Sun S., Zhao M., Cui J., Liu D. (2021). Relationship of Problematic Smartphone Use, Sleep Quality, and Daytime Fatigue Among Quarantined Medical Students During the COVID-19 Pandemic. Front. Psychiatry.

[48] Líška D., Liptaková E., Babičová A., Batalik L., Baňárová P.S., Dobrodenková S. (2022). What is the quality of life in patients with long COVID compared to a healthy control group?. Front. Public Health.

[49] Margalit I., Yelin D., Sagi M., Rahat M.M., Sheena L., Mizrahi N., Yahav D. (2022). Risk Factors and Multidimensional Assessment of Long Coronavirus Disease Fatigue: A Nested Case-Control Study. Clin. Infect. Dis..

[50] Sandler C.X., Wyller V.B., Moss-Morris R., Buchwald D., Crawley E., Hautvast J., Lloyd A.R. (2021). Long COVID and Post-infective Fatigue Syndrome: A Review. Open Forum Infect. Dis..

[51] Twomey R., DeMars J., Franklin K., Culos-Reed S.N., Weatherald J., Wrightson J.G. (2022). Chronic Fatigue and Postexertional Malaise in People Living With Long COVID: An Observational Study. Phys. Ther..

[52] Vélez-Santamaría R., Fernández-Solana J., Méndez-López F., Domínguez-García M., González-Bernal J.J., Magallón-Botaya R., Santamaría-Peláez M. (2023). Functionality, physical activity, fatigue and quality of life in patients with acute COVID-19 and Long COVID infection. Sci. Rep..

